# Transcriptome and Volatilome Analysis During Growth of *Brochothrix thermosphacta* in Food: Role of Food Substrate and Strain Specificity for the Expression of Spoilage Functions

**DOI:** 10.3389/fmicb.2019.02527

**Published:** 2019-11-08

**Authors:** Nassima Illikoud, Rodérick Gohier, Dalal Werner, Célia Barrachina, David Roche, Emmanuel Jaffrès, Monique Zagorec

**Affiliations:** ^1^SECALIM, INRA, Oniris, Nantes, France; ^2^Aérial, ITAI-CRT, Illkirch, France; ^3^MGX, CNRS, INSERM, University of Montpellier, Montpellier, France; ^4^Génomique Métabolique, Génoscope, Institut François Jacob, CEA, CNRS, Université d’Evry, Université Paris-Saclay, Evry, France

**Keywords:** shrimp, meat, transcriptome, volatile organic compounds, diacetyl, acetoin, food spoilage, *Brochothrix thermosphacta*

## Abstract

*Brochothrix thermosphacta* is one of the main spoilers in food, responsible for meat and seafood spoilage through the production of malodorous volatile organic compounds. The molecules produced by this bacterium depend on the substrate (meat or seafood) and the storage conditions such as gas mixtures used in the packaging. It seems also that the spoilage potential is strain dependent as production of diacetyl and acetoin, two molecules responsible for seafood spoilage, varies with strains. Therefore, this suggests the involvement of different metabolic functions depending on both food substrate and strain capacities. In this study, we selected two strains with different abilities to produce diacetyl and acetoin and compared their behavior after grown in beef or cooked peeled shrimp juices. We determined the genes upregulated by both strains depending on the growth substrate and those that were specifically upregulated in only one strain. The genes upregulated by both strains in meat or in shrimp juice revealed the importance of the substrate for inducing specific metabolic pathways. The examination of genes that were specifically upregulated in only one of the two strains revealed strain features associated to specific substrates and also strain-specific regulations of metabolic pathways putatively leading to different levels of spoilage molecule production. This shows that the spoilage potential of *B. thermosphacta* depends on nutrients provided by food substrate and on metabolic activity potential that each strain possesses.

## Introduction

*Brochothrix thermosphacta* is commonly reported as belonging to the microbiota of a wide range of food matrices including various raw and processed meat and seafood products (Chaillou et al., 2015; Remenant et al., 2015; Illikoud et al., 2018a). This results from its ubiquitous nature and its ability to grow at the refrigerated temperatures in various atmosphere packaging used for food storage (Stackebrandt and Jones, 2006). *B. thermosphacta* has been frequently associated to food spoilage through the production of volatile organic compounds (VOCs) causing off-odors (Casaburi et al., 2014, 2015; Odeyemi et al., 2018). The production of such molecules seems to be food matrix dependent (Illikoud et al., 2018a). For example, acetoin, diacetyl, and 3-methylbutanol associated with cheesy and creamy dairy off-odors have been reported in beef meat (Dainty et al., 1989; Casaburi et al., 2014), while in cooked and peeled shrimp, diacetyl and 3-methyl-1-butanal were produced and responsible for strong butter, buttermilk-like, sour, and nauseous off-odors (Jaffrès et al., 2011). Recently, genotypic and phenotypic analysis of a *B. thermosphacta* strain collection revealed that genetic diversity was not related to strain ecological origin (Illikoud et al., 2019). The amounts of acetoin and diacetyl produced were variable among strains, but also depend on the type of food matrix used to grow bacteria. Hence, these results suggest that the spoilage potential of *B. thermosphacta* may be both strain- and food matrix-dependent. Genome comparison showed a high degree of similarity in the gene repertoire of *B. thermosphacta* strains (Stanborough et al., 2017; Illikoud et al., 2018b). The major differences in the gene content consisted of phages and plasmids, or genes encoding cell surface proteins or adhesins, which could not explain the different spoilage abilities of the strains but rather suggested a variable fitness for growing in different ecological niches. Nevertheless, different base substitutions in or upstream from genes encoding enzymes required for the production of spoilage molecules were noticed when comparing several *B. thermosphacta* genomes (Illikoud et al., 2018b). This suggests that different spoilage abilities among strains may rely on differences in gene expression or enzyme activity levels. The characterization of the genes expressed and the metabolites produced by *B. thermosphacta* strains in different food matrices should contribute to better understand the *B. thermosphacta* spoilage mechanisms and the reasons for spoilage potential differences. Therefore, our strategy for the present study was to compare volatilomes and transcriptomes of two different strains inoculated in two model matrices (meat and shrimp juices). Our objective was to discriminate the strain effect from the matrix effect by searching the genes differentially expressed depending on the matrix and on the strain. We also attempted to identify metabolic pathways involved in the spoilage potential of *B. thermosphacta* in meat and in shrimp.

## Materials and Methods

### Bacterial Strains, Media, and Growth Conditions

*Brochothrix thermosphacta* CD 337 and TAP 175 (Illikoud et al., 2018b) isolated from spoiled cooked peeled shrimp and non-spoiled chicken cuts, respectively, were used in this study. The strains were stored at −80°C in Brain Heart Infusion (BHI) broth (VWR, France) added with 20% (v/v) glycerol and were routinely grown at 25°C in BHI medium.

Bacterial enumeration were performed after serial dilutions and plating on Plate Count Agar (PCA) (Biomerieux, France) for total viable counts and on selective STAA agar base containing STAA selective supplement (Oxoid, France) for *B. thermosphacta*, after incubation for 48 h at 30 and 25°C, respectively.

### Meat and Shrimp Juice Preparation

Meat and shrimp juices were prepared as previously described (Illikoud et al., 2019) with slight modifications as described below. Ground beef (25 g) purchased from a local supermarket was stomached in 100 ml of Ringer’s solution (Oxoid, France) and the mixture was centrifuged at 6000 × *g* for 20 min at 4°C. The supernatant was then filtered through a 0.45-μm membrane filter before sterilization with a 0.2-μm membrane filter. Cooked peeled shrimp juice was prepared by crushing 100 g of frozen raw peeled shrimps from Ecuador (91/100 without sulfite, purchased from industry, Nantes, France) in 300 ml of sterile distilled water. The mixture was heated (100°C; 2 min) and centrifuged at 600 × *g* for 20 min, and the supernatant was autoclaved (100°C; 30 min). These juices, mimicking raw ground beef and cooked peeled shrimp, were aliquoted by 20 ml in 45-ml sterile glass vials and then stored frozen at −80°C until use.

### Challenge Tests

*B. thermosphacta* strains were harvested from overnight cultures (4 ml) by centrifugation (13,400 × *g*, 5 min, 4°C), washed with 1 ml of Ringer’s solution, centrifuged with the same parameters, resuspended in 1 ml of meat or shrimp juice, and inoculated in 20 ml of food juice at an initial concentration of 10^6^ CFU/ml. Glass vials were closed and inoculated juices were incubated at 8°C. Those conditions generated a microaerophilic environment during storage. Non-inoculated juice samples were included as controls. Bacterial enumeration, volatilome analysis, and pH measurement (Crison pH-meter) were performed at day 0, 1, 2, 4, and 7, whereas transcriptome analysis was performed at day 4. Challenge tests were performed in triplicate.

### VOC Analysis

At each sampling time, vials were immediately frozen at −20°C without opening the vials and stored frozen until analysis. For VOC analysis, frozen vials were thawed overnight at room temperature. Nine milliliters of sample extract was transferred into a 20-ml headspace vial and sealed with a screwcap with silicone rubber septum. Analyses were carried out in triplicate.

Volatile organic compounds were determined by HS–GC–MS. All analyses were performed on a Varian 450 gas chromatograph (Varian, Palo Alto, CA, United States) coupled to a Varian 225-IT mass spectrometer (Varian), equipped with a CTC Combi PAL (CTC Analytics AG, Zwingen, Switzerland). Samples were equilibrated by agitation at 60°C for 20 min prior to injection and 1 ml was drawn out from the head space to inject in the GC. The HS-GC–MS conditions were as follows. Capillary column: DB-624 UI (30 m × 0.25 mm I.D × 1.4 mm film thickness) (Agilent Technologies); carrier gas: helium with a flow rate of 1.4 ml/min; injection port mode: splitless; needle temperature: 60°C; injection temperature: 220°C. The oven temperature was programed from an initial temperature of 40°C (7 min holding), rising to 50°C at 4°C/min (1 min holding), to 70°C at 4°C/min (1 min holding), to 120°C at 3°C/min (2 min holding), and to 245°C at 30°C/min (4 min holding). Transfer line temperature was 250°C. The temperatures of the manifold and the ion trap were kept constant at 150 and 40°C, respectively. Results were obtained in scan mode at 4 scans/s in the mass range (m/z) of 35–350 atomic mass units. VOCs were identified by comparison of GC retention times and mass spectra with those of the standard compounds (LGC Standards, France; Techlab, France and Cluzeau Info Labo, France). These standard compounds were selected based on a review of published data of VOCs detected in meat and seafood spoiled in the presence of *B. thermosphacta* (Illikoud et al., 2018a). Peak area (in arbitrary unit) was used as quantitative data to monitor the relative changes of VOCs over storage and, where appropriate, to correlate these findings with bacterial strain as well as with the studied food matrices. This relative quantification was scored from - (no detection) to +++ (highest detection).

### RNA Preparation and Sequencing

At day 4, 1 ml of the inoculated juices was sampled and bacteria were harvested by centrifugation at 10,000 × *g* for 5 min at 4°C. The cell pellet was immediately resuspended in 200 μl of RNA Protect Cell Reagent (Qiagen, Courtaboeuf, France) and frozen at −80°C. Total RNA was extracted from the pellet using the All Prep Qiagen Kit following the manufacturer’s instructions: bacteria were first chemically lysed in phenol/chloroform and Qiagen lysis buffer containing β-mercaptoethanol, and mechanical lysis was carried out using a FastPrep (MP Biomedicals, Illkirch, France) for 40 s at a frequency of 5.5 m/s. RNA were then purified by loading on RNeasy^®^ spin column, followed by washing and elution. Total RNAs were quantified using a Nanodrop 2000 spectrophotometer (Thermo Scientific, Illkirch, France) and quality was checked by Experion^TM^ Automated Electrophoresis System (Biorad, Marnes-la-Coquette, France). rRNA depletion was performed on 1 μg total RNA with the Ribo-Zero rRNA Removal Kit dedicated to bacteria (Illumina, San Diego, CA, United States). Purified mRNA quality was validated by capillary electrophoresis on a Fragment Analyzer (Advanced Analytical, Ankeny, IA, United States).

RNA-Seq libraries were constructed with the Truseq stranded mRNA sample preparation (low-throughput protocol) kit from Illumina. Purified mRNA (10 ng) were cleaved into small fragments using divalent cations under elevated temperature. The cleaved RNA fragments were converted into a first cDNA strand using SuperScript II reverse transcriptase (Invitrogen), actinomycin D, and random hexamer primers. A second cDNA strand was synthesized by replacing dTTP with dUTP. These cDNA fragments then have the addition of a single “A” base and subsequent ligation of the adapter. The products were then purified and enriched with 15 PCR cycles. The final cDNA libraries were validated with a Fragment Analyzer (Advanced Analytical, Ankeny, IA, United States) and quantified with a KAPA qPCR kit (Kapa Biosystems, Wilmington, MA, United States). On one sequencing lane of a flowcell V4, the 12 libraries (2 strains × 2 juices × 3 replicates) were pooled in equal proportions, denatured with NaOH, and diluted to 12 pM before clustering.

Cluster formation, primer hybridization and single-end read, and 50-cycle sequencing were performed on cBot and HiSeq2500 (Illumina, San Diego, CA, United States), respectively. Image analysis and base calling were performed using the HiSeq Control Software and Real-Time Analysis component. Demultiplexing was performed using Illumina’s sequencing analysis software. The quality of the data was assessed using FastQC from the Babraham Institute and the Illumina software SAV (Sequence Analysis Viewer).

FastqQ Screen (Wingett and Andrews, 2018) has been used to search sequences from commonly sequenced genomes in the facility that produced the data, and potential contamination sources: *Homo sapiens*, *Mus musculus*, *Drosophila melanogaster*, *Arabidopsis thaliana*, *Bos taurus*, *Gallus gallus*, *Caenorhabditis elegans*, *Saccharomyces cerevisiae*, *Candida albicans*, *Aspergillus niger*, *Escherichia coli*, *Streptococcus pyogenes*, *Bacillus cereus*, *Chlamydia trachomatis*, *Enterococcus faecalis*, *Propionibacterium acnes*, *Staphylococcus aureus*, a set of *Mycoplasma*, and a set of rRNA from different species. The PhiX genome (used as illumina control sequences) and illumina adapters (TruSeq and Nextera) have also been searched.

### Transcriptome Analysis

Transcriptomic high-throughput sequencing data were analyzed using a bioinformatic pipeline implemented in the Microscope platform (Médigue et al., 2017). The current pipeline is a “Master” shell script that launches the various parts of the analysis (i.e., a collection of Shell/Perl/R scripts) and controls for all tasks having been completed without errors. In a first step, the RNA-Seq data quality was assessed by including options like read trimming or merging/split paired-end reads. In a second step, the reads of each sample were mapped against the corresponding *B. thermosphacta* genome sequences: CD337 complete genome sequence ERZ500814 and TAP175 draft genome sequence ERZ500815 (Illikoud et al., 2018b), using the SSAHA2 package (Ning et al., 2001). This latter combines the SSAHA searching algorithm (sequence information is encoded in a perfect hash function), aiming at identifying regions of high similarity, and the cross-match sequence alignment program (Ewing et al., 1998), which aligns these regions using a banded Smith–Waterman–Gotoh algorithm (Smith and Waterman, 1981). An alignment score equal to at least half of the read is required for a hit to be retained. To reduce the false-positive discovery rate, the SAMtools (v.0.1.8) (Li et al., 2009) was then used to extract reliable alignments from SAM formatted files. The number of reads matching each genomic object harbored by the reference genome was subsequently computed with the Bioconductor-GenomicFeatures package (Carlson et al., 2011). If reads matched several genomic objects, the count number was weighted in order to keep the same total number of reads. Finally, the Bioconductor-DESeq package (Anders and Huber, 2010) with default parameters was used to analyze raw count data and test for differential expression between conditions.

### Data Availability

The data have been submitted to the Sequence Read Archive database, with BioProject accession number PRJNA546512.

## Results and Discussion

### Bacterial Growth in Food Juices

**FIGURE 1:**
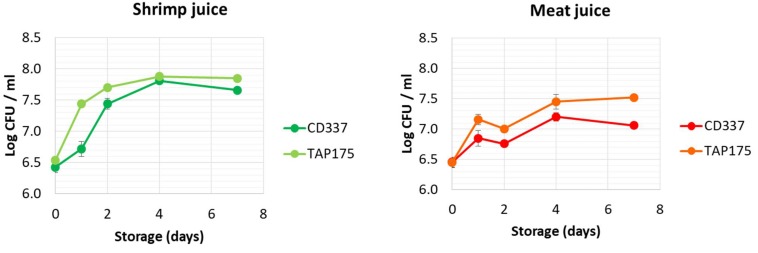
Growth kinetics of *Brochothrix thermosphacta* CD 337 and TAP 175 in meat and shrimp juices monitored in STAA-specific medium.

Growth of *B. thermosphacta* CD 337 and TAP 175 in beef and shrimp juices was monitored during storage at 8°C for 7 days (Figure 1). Total aerobic mesophilic and *B. thermosphacta* counts were similar (data not shown). The absence of bacteria detection (<1 CFU/ml) in the non-inoculated control juices throughout the storage confirmed that both juices were sterile before inoculation (data not shown). As observed in Figure 1, the two strains grew in both juices, reaching stationary phase after 2–4 days of storage. In shrimp juice, *B. thermosphacta* population increased rapidly during the first 2 days and reached a final cell density of 7.8–8.0 log CFU/ml after 4 days. In meat juice, the stationary phase was reached at day 4 but bacterial population did not exceed 7.5 log CFU/ml for both strains. Initial growth rate of *B. thermosphacta* TAP 175 was slightly higher than that of strain CD 337 in both media.

### pH Evolution During Storage

**FIGURE 2:**
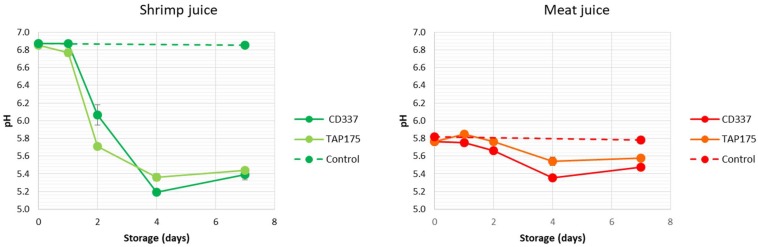
Evolution of pH during storage at 8°C of meat and shrimp juices inoculated by *B. thermosphacta* strains CD 337 and TAP 175.

The initial pH values were 6.8 and 5.8 in shrimp and meat juices, respectively (Figure 2). The pH of control samples was constant along storage, whereas it decreased in inoculated juices. No significant difference was observed between strains. The pH of shrimp juice decreased to 5.2–5.4 after 4 days of storage, whereas that of meat juice decreased to 5.4–5.6 at day 4. Then, pH remained stable or slightly increased between day 4 and day 7.

### VOCs Production

Seven compounds could be detected and identified (Table 1), among which 2-ethyl-1-hexanol present at similar levels in both control and inoculated meat juice and therefore considered as issued from beef meat and independent of bacterial metabolism. VOC production varied depending on the matrix. In meat juice, only ethanol and acetoin were detected after inoculation of *B. thermosphacta*, whereas six compounds (ethanol, isobutyraldehyde, 2-methylbutyraldehyde, 3-methylbutyraldehyde, acetoin, and 3-methyl-1-butanol) were produced in inoculated shrimp juices. Slight differences between the two strains were observed, mostly in the production kinetics. For example, 3-methyl-1-butanol was detected at days 4 and 7 in shrimp juice inoculated with CD 337 while it was detected only at day 7 in the juice inoculated by TAP 175 (Table 1).

**TABLE 1:** Volatile organic compounds (VOCs) identified in meat and shrimp juices.

	**VOCs**	**Control^a^**	**CD 337**				**TAP 175**			
			D1^b^	D2	D4	D7	D1	D2	D4	D7
Shrimp	Ethanol	−	−	+	++	++	−	+	++	++
juice	Isobutyraldehyde	−	−	−	+	−	−	+	−	−
	3-Methylbutyraldehyde	−	+	++	+++	+	+	++	++	+
	2-Methylbutyraldehyde	−	+	++	++	−	+	++	++	−
	Acetoin	−	−	−	−	+	−	−	−	−
	3-Methyl-1-butanol	−	−	−	+	++	−	−	−	+
	2-Ethyl-1-hexanol	−	−	−	−	−	−	−	−	−
Meat	Ethanol	−	−	−	+	+	−	−	+	++
juice	Isobutyraldehyde	−	−	−	−	−	−	−	−	−
	3-Methylbutyraldehyde	−	−	−	−	−	−	−	−	−
	2-Methylbutyraldehyde	−	−	−	−	−	−	−	−	−
	Acetoin	−	−	−	+	+	−	−	+	++
	3-Methyl-1-butanol	−	−	−	−	−	−	−	−	−
	2-Ethyl-1-hexanol	+	+	+	+	+	+	+	+	+

*^a^Values were obtained from non-inoculated juices incubated at 8°C for 0, 1, 2, 4, and 7 days. ^b^In juices inoculated with *Brochothrix thermosphacta* CD 337 or TAP 175, values are given after 1, 2, 4, and 7 days of growth at 8°C.*

### Characterization of Differentially Expressed Genes

RNAs were extracted at day 4, i.e., approximately when strains reached the stationary phase and produced various VOCs. Cells were thus considered metabolically active.

A total of 248,189,013 reads were generated using HiSeq 2500 single-end (Illumina, San Diego, CA, United States). For each transcriptome, more than 98% of the generated reads had a PHRED quality score higher than 30, meaning that the reads were of high quality (i.e., base call accuracy higher than 99.9%). Summary of data is given in Supplementary Table S1. We searched for genes whose expression [log2 fold change (LFC_2_) > 2; adjusted *p*-value (FDR) < 0.05] was increased during growth in meat compared to shrimp juice. We focused first on the genes differentially expressed by both strains and then on those whose expression varied in only one of the two strains. We performed similarly for genes that were specifically upregulated in shrimp compared to beef juice. This enabled us to discriminate the strain effect from the matrix effect on the differential expression of genes. Functions and metabolic pathways putatively inducted during growth in meat or shrimp juice were then deduced from the manually cured annotated genomes of both strains (Illikoud et al., 2018b).

### Genes Specifically Upregulated in Meat Juice

Among the genes upregulated in meat juice (*N* = 97 in CD 337 and *N* = 161 in TAP 175), 63 were common to both strains. Out of those, eight encoded proteins of unknown function; nine were annotated as encoding putative enzymes, transporters, or regulators of unknown specificity; and two were related to ribosomal proteins or RNAs. The remaining 44 genes could be correlated to functions that are associated to growth on meat substrate (Supplementary Table S2).

**FIGURE 3:**
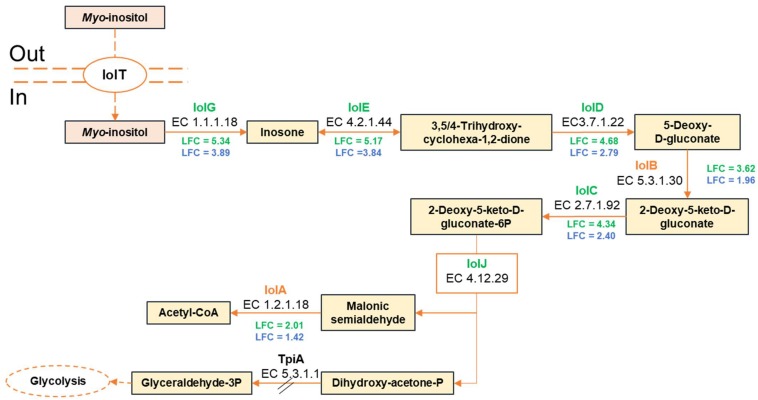
Predicted pathway of *myo*-inositol catabolism by *B. thermosphacta* strains CD 337 and TAP 175 in meat juice. LFC values were represented with a color code depending on strain: CD 337 (green); TAP 175 (blue).

For example, 9 of the 11-gene cluster *iolRABCDETGHIJ* involved in *myo*-inositol utilization were upregulated by both strains. Nevertheless, the *iolAB* genes were upregulated in CD 337 only with LFC_2_ values of 2.01 and 3.62, respectively, whereas in TAP 175, these values were below the used cutoff (1.42 and 1.96, respectively), suggesting less efficient *myo*-inositol utilization in this strain. The *iol* gene cluster encodes the transcriptional regulator IolR, the transporter IolT, and the enzymes responsible for catabolism of inositol as a carbon source. This compound is present not only in foods of vegetable origin (Clements and Darnell, 1980), but also in meat (Lilyblade and Peterson, 1962) from which it was first purified (Hoffmann-Ostenhof and Pittner, 1982). Several microorganisms can use *myo*-inositol as carbon source. These include *Salmonella*, *Serratia*, *Klebsiella*, and *Bacillus subtilis* (Legakis et al., 1976; Yoshida et al., 1997; Kröger and Fuchs, 2009). In *B. subtilis*, IolR acts as a transcriptional repressor in the absence of inositol. *Myo*-inositol acts as an inducer antagonizing IolR binding, enabling then the expression of the *iol* genes (Yoshida et al., 1999, 2002). The same regulation might thus also exist in *B. thermosphacta.* First, *myo*-inositol is taken into the cell by IolT and then metabolized by IolGEDBCJ enzymes to malonic semialdehyde and dihydroxyacetone phosphate (Yoshida et al., 2004; Figure 3). Malonic semialdehyde could then be metabolized into acetyl-CoA by IolA. Further metabolism of dihydroxyacetone phosphate is unclear from the transcriptome results. Indeed, its conversion into glyceraldehyde-3-phosphate by the triose phosphate isomerase (TpiA) might be impaired since *tpiA* was downregulated in meat juice.

**FIGURE 4:**
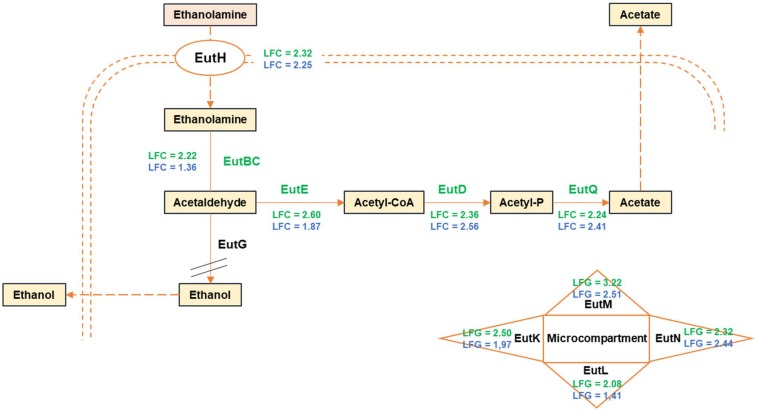
Proposed pathway of ethanolamine utilization by *B. thermosphacta* strains CD 337 and TAP 175 in meat juice. LFC values were represented with a color code depending on strain: CD 337 (green); TAP 175 (blue). The gene encoding EutG was downregulated in meat juice. EutKLMN form a micro compartment encasing the enzymes necessary for ethanolamine catabolism.

Part of the *eut* gene cluster involved in ethanolamine utilization was also upregulated in meat juice by both strains (Figure 4). Ethanolamine is a major constituent of lipids of eukaryotic cells thus present in beef meat. It is also present in fish but at lower levels (Fogerty et al., 1991). Several bacteria can use ethanolamine as a unique source of carbon and/or nitrogen, but the gene content, organization, and regulation of the *eut* operon are highly variable between bacterial species (Kaval and Garsin, 2018). The *B. thermosphacta eut* gene cluster [*eutP-pduO-eutGSVWABCLKEMD-(euthyp)-NHQ*] was similar to that of *Enterococcus faecalis*, i.e., encompassed *eutV* and *eutW* genes encoding a two-component regulatory system, and gene order was similar, except for the presence of an *euthyp* gene observed in *B. thermosphacta* and in other bacterial species, but absent from that of *E. faecalis*. In meat, *eutCLKEMDNHQ* were upregulated in CD337, whereas *eutB* LFC_2_ was below the used threshold (1.34), and only *eutMDNHQ* were upregulated in TAP175. Nevertheless, *eutK* was also considered as upregulated in TAP175 with an LFC_2_ value of 1.97 just below the threshold used. Those of *eutL* and *eutC* were, respectively, 1.41 and 1.36. Intriguingly, *eutA* was not expressed and *eutG* was downregulated. It seems, however, that *B. thermosphacta* could indeed use ethanolamine in meat as shown in Figure 4 since all the necessary elements were upregulated. EutKMNL proteins form a micro compartment encasing the enzymes necessary for ethanolamine catabolism (Sagermann et al., 2009; Takenoya et al., 2010). Ethanolamine, after transport by EutH, is converted into acetaldehyde and ammonia by EutBC (the two subunits of cobalamin-dependent ethanolamine ammonia lyase). EutA is necessary for reactivating EutBC unless adenosylcobalamin is present (Roof and Roth, 1989). Since adenosylcobalamin is known to be the major form of vitamin B12 present in beef meat (Czerwonka et al., 2014), one can hypothesize that the lack of EutA did not impair ethanolamine catabolism. Acetaldehyde is then converted to acetyl-CoA by EutE (acetaldehyde dehydrogenase) and acetyl-phosphate by a phosphotransacetylase EutD (Kaval and Garsin, 2018). Acetyl-phosphate can then be converted to acetate by EutQ (Kaval and Garsin, 2018). Acetaldehyde can also be converted to ethanol by EutG (an alcohol dehydrogenase) (Del Papa and Perego, 2008; Pitts et al., 2012). However, since *eutG* was downregulated in meat juice, this suggests that *B. thermosphacta* catabolized ethanolamine into acetate rather than ethanol.

A gene cluster encompassing *ascRsnaAtcyJKLM* involved in sulfur compound utilization and a putative FMN reductase related with sulfur starvation were also upregulated in meat juice. AscR is a transcriptional regulator and *snaA* is encoding an *N*-acetyltransferase acting on sulfur. *tcyKJLM* are involved in cysteine uptake. *tcyK* LFC_2_ values were 2.11 in CD337 and 1.83 in TAP175, suggesting that all the system was upregulated in both strains. In *B. subtilis*, the expression of *snaA* was reported to be strongly increased in the presence of glutathione (Coppée et al., 2001) a compound present at higher concentrations in beef meat than in raw fish (Rose et al., 1999). This most probably explains the upregulation of this set of genes to cope with sulfur starvation and/or presence of glutathione in beef juice.

The five genes of the pot operon (*potRABCD*) involved in spermidine/putrescine uptake were upregulated in meat juice. Seiler (1999) reported that these polyamines and their intermediates are found in a wide range of organisms including bacteria, plants, and animals. They are essential for bacterial growth. Several studies indicate that polyamine transporters are highly conserved in many Gram-positive and Gram-negative bacteria such as *Escherichia coli*, *Salmonella enterica*, and *S. aureus* (Igarashi and Kashiwagi, 1999). These systems were described to be involved in adaptation and/or survival of microorganisms and in biofilm formation (Wortham et al., 2007; Shah and Swiatlo, 2008; Shah et al., 2011). In bacteria, polyamine uptake from the environment may provide resistance to various environmental stresses including temperature change, osmotic pressure, reactive oxygen species, or other toxic compounds (Gevrekci, 2017). This suggests that these polyamines may be transported by *B. thermosphacta* and contribute to its survival in meat. However, as in *S. aureus*, the preferred substrates of the Pot system were spermidine and spermine, but not putrescine (Yao and Lu, 2014), we cannot ensure which substrate is transported by the PotABCD transporter in *B. thermosphacta*.

A pyruvate uptake transporter encoded by *pftAB* genes was also upregulated in meat. Pyruvate has been reported to be one of the substrates used by spoilage bacteria in meat. It is a precursor of various spoilage molecules such as acetoin, diacetyl, and acetic acid (Nychas et al., 2007; Casaburi et al., 2015).

**FIGURE 5:**
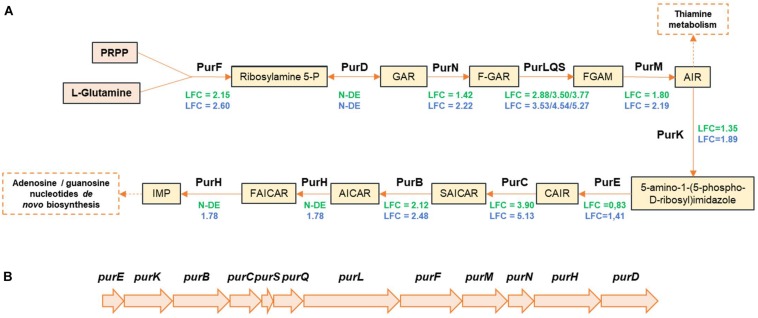
Proposed pathway of purine biosynthesis in meat juice by *B. thermosphacta* strains CD 337 and TAP 175 **(A)**, and *pur* operon organization **(B)**. LFC values were represented with a color code depending on strain: CD 337 (green); TAP 175 (blue). Abbreviations: N-DE, not differentially expressed; PRPP, 5-phosphoribosylpyrophosphate; GAR, glycinamide ribonucleotide; FGAR, formylglycinamide ribonucleotide; FGAM, formylglycinamidine ribonucleotide; AIR, aminoimidazole ribonucleotide; CAIR, carboxyaminoimidazole ribonucleotide; SAICAR, *N*-succinocarboxamide-5-aminoimidazole ribonucleotide; AICAR, aminoimidazole-4-carboxamide ribonucleotide; FAICAR, 5-formamido-4-imidazolecarboxamide ribonucleotide; IMP, inosine monophosphate.

Part of the *pur* operon (*purBCSQLF*) involved in purine biosynthesis was upregulated in both strains. *purM* and *purN* were also upregulated in TAP175, whereas their LFC_2_ values in CD337 were below the threshold (1.80 and 1.42, respectively). The purine biosynthetic pathway is conserved in bacteria (Kappock et al., 2000; Zhang et al., 2008). Inosine monophosphate (IMP) is the first nucleotide formed from purine biosynthesis. Then, IMP may be converted into adenosine monophosphate (AMP) and/or guanosine monophosphate (GMP) (Figure 5A). In CD 337 and TAP175, respectively, *purHD* and *purD* were not found among the differentially expressed genes. One can hypothesize that since these two genes are at the 3′-end of the operon (Figure 5B), their RNAs might be degraded and thus not appearing as upregulated in our experiments. The genes *xapB-pupG* and *pyrRP* encoding permeases for xanthine and uracil and their corresponding phosphoribosyltransferase converting them into xanthine monophosphate (XMP) and uridine monophosphate (UMP) were also upregulated in the two strains. A gene encoding a putative glutamine amidotransferase potentially converting XMP into GMP was specifically upregulated in TAP175 (LFC_2_ = 2.87), whereas its LFC_2_ value in CD337 was 1.93. Moreover, three other genes encoding putative guanine, hypoxanthine, or xanthine permeases were differently upregulated in the two strains: LFC_2_ values of *pbuG*, *pbuX*, and *pbuO-*like genes were 3.33 *vs.* 1.92, 2.24 *vs.* 1.33, and 1.78 *vs.* 2 in TAP175 and CD337, respectively. This suggests that *B. thermosphacta* purine and pyrimidine metabolism in meat may result from both uptake from the matrix and *de novo* synthesis and that the two strains cope differently with nucleobase transport and synthesis by activating different systems.

Finally, a five-gene cluster encoding the three subunits of a putative Fe^3+^ (ferric ion) transport system, a downstream two-component regulatory system, and on the opposite orientation a putative Na+/H+ antiporter were also upregulated by both *B. thermosphacta* strains in meat juice. Ferric iron form is available in raw meat (Carpenter and Clark, 1995). Intracellular iron is used in biological reactions such as oxygen transport, gene regulation, DNA biosynthesis, and reparation (Andrews et al., 2003). Moreover, *B. thermosphacta* has been reported to require iron for growth under aerobic conditions (Thomson and Collins-Thompson, 1986).

In addition to the abovementioned genes, several were upregulated after growth in meat juice in only one of the two strains. Among those, 25 were specifically upregulated in CD337, with 6 being of unknown function and absent from the TAP175 chromosome (Supplementary Table S2). Thirteen were of unknown or uncertain function but the six remaining composed the tryptophan biosynthesis operon (*trpABFCGDE*), indicating a different metabolism between the two strains, although not reliable to meat spoilage.

In TAP 175, in addition to some described above, 95 genes were upregulated after growth in meat only in this strain, with a majority (*N* = 51) of unknown function. Twelve were associated to transport (*N* = 8) or regulatory (*N* = 4) functions with unknown specificity, and 13 were associated to RNA metabolism (including 4 tRNA genes and 5 miscellaneous RNA, and 4 to DNA). A gene encoding a flavoprotein annotated as putative phosphopantothenoylcysteine decarboxylase, which was absent in CD337, and a gene encoding a putative acetyl transferase were upregulated in TAP175 but could not be related to any functional metabolic pathway.

Nevertheless, several genes specifically upregulated in TAP175 could be informative about functions or conditions associated to growth in meat. Among those, three genes encoding a putative alkaline-shock protein, a cold-shock protein (*cspC*), and a free methionine sulfoxide reductase (*msrC*), all involved in stress response, were upregulated in TAP175, whereas in CD337, *cspC* was not expressed and the upregulation of the other was below the threshold (LFC_2_ being 1.21 and 1.28). A PTS-dependent transporter, specific for beta-glucosides, was upregulated in TAP175 (LFC_2_ = 1.83 in CD337).

The *acpA* gene encoding an acyl carrier protein involved in fatty acid biosynthesis was upregulated in TAP175 (LFC_2_ = 2.24 *vs.* 1.79 in CD337). Nevertheless, although located downstream from a cluster of genes involved in lipid synthesis as in *B. subtilis* (Martinez et al., 2010), only *acpA* was upregulated. Two type I signal peptidases (SipA and SipS) similar to the SipS protein of *B. subtilis* responsible for the processing of precursors of secreted protein (Bolhuis et al., 1996) were also upregulated only in TAP175 although their homologs exist in the CD337 genome. Also, *lspA* encoding the type II signal peptidase for lipoprotein processing (Tjalsma et al., 1999) was upregulated. *yrbF* encoding the auxiliary subunit of the preprotein translocase was upregulated. In *Lactobacillus buchneri*, this protein has been reported as upregulated as a response to ethanol (Liu et al., 2016). A gene encoding a putative CDP-glycerol glycerophosphotransferase described as involved in teichoic acid (poly-glycerol) biosynthesis was specifically upregulated by TAP175. It should be noticed that among the 51 proteins of unknown function whose genes were upregulated in TAP175, 14 were annotated as membrane associated or exported. Therefore, one can hypothesize that, when grown in meat, this strain may specifically produce proteins either secreted or associated to the cell surface. Indeed, the importance of cell surface composition for interactions of *B. thermosphacta* with the different environments it can grow in has been suspected (Stanborough et al., 2017, 2018; Illikoud et al., 2018b).

### Genes Specifically Upregulated in Shrimp Juice

Among the 74 genes upregulated in both strains after growth in shrimp juice compared to meat juice (Supplementary Table S2), 11 coded for proteins of unknown function, 8 coded for putative enzymes, and 1 coded for a transporter (*vlmR*), all of unknown specificity. Five were tRNA genes and four encoded miscellaneous RNA including the 6S RNA. This may play a role later in transcriptional regulation. Indeed, in *E. coli* for example, 6S RNA has been reported as accumulating during stationary phase (Cavanagh et al., 2012), downregulating transcription, and important for modulating stress and survival during nutrient limitation (Cavanagh and Wassarman, 2014). This correlates with the sampling time of the present study for RNA extraction (at day 4), when bacterial growth ceased in shrimp juice.

Two putative transporters ComEA and ComF involved in DNA uptake were upregulated (CD 337 LFC_2_ = 2.34 and 2.01, respectively; TAP 175 LFC_2_ = 3.35 and 2.51, respectively). In *Thermus thermophilus*, the *comEA* operon is upregulated by nutrient limitation and low temperature (Salzer et al., 2016). In addition, a putative *comGC* gene was upregulated in TAP175, whereas it was not expressed in CD337. ComGC is a pilin that can be involved in various cell surface functions such as DNA transport, biofilm formation, or adhesion (Muschiol et al., 2017).

Interestingly, the two genes *copA* and *copZ* encoding a copper exporter system were also upregulated in shrimp juice (CD 337 LFC_2_ = 4.32 and 2.57, respectively; TAP 175 LFC_2_ = 6.24 and 3.27, respectively). Copper is present in crustaceans as these organisms contain the respiratory protein hemocyanin, a hexamer with two Cu^2+^ ions per subunit (Marxen et al., 2013). Copper content ranges from ∼0.1 to ∼1 mg/kg in meat (Cabrera et al., 2010) but is higher in shrimp, with an average value of ∼20 mg/kg and the lowest concentration of ∼4 mg/kg (Olmedo et al., 2013; Adams et al., 2015). Copper is essential for bacterial growth since it is a required cofactor for a number of enzymes with oxidase or oxygenase activities, detoxification of oxygen-derived free radicals, and electron transfer (Frausto da Silva and Williams, 2001). However, at high concentrations, it can be toxic due to its interaction with proteins, enzymes, nucleic acids, and metabolites (Trevors and Cotter, 1990). In *B. subtilis*, CopZ contributes to the copper sequestration and interacts with CopA for copper export (Radford et al., 2003). Although the copper content and availability in our experimental juices have not been measured, one can hypothesize that an excess of copper in shrimp juice, compared to meat juice, most probably induced its export out of the cells.

Five genes encoding proteins related to stress response or chaperoning activity were upregulated in both strains: GroEL, HrcA, GrpE, DnaK, and a Gls24 homolog. *clpB* was also upregulated in both strains although the gene was split into two fragments in CD337. In CD337, *groES* was also upregulated whereas its LFC_2_ was only 1.41 in TAP175. Conversely, ClpC and DnaJ were upregulated only in TAP175 (LFC_2_ = 2.41 and 2.54, *vs.* 1.79 and 1.96, respectively, in CD337). ClpB, a stress-induced multi-chaperone system with DnaK, DnaJ, and GrpE, has been described to be involved in the processing of protein aggregates and/or damaged proteins in *B. subtilis* (Mogk et al., 1997) and *Listeria monocytogenes* (Liu et al., 2002). This system has been also found in lactic acid bacteria (Papadimitriou et al., 2016). It seems thus that the machinery for refolding injured proteins is upregulated in *B. thermosphacta* in shrimp juice.

A large proportion of genes upregulated after growth in shrimp juice encode proteins involved in various sugar transport and/or utilization. These encompassed a response common to both strains but also genes whose response was strain specific. Among genes whose upregulation was shared by both strains, 10 were involved in transport/phosphorylation of carbon sources, in particular several phosphoenolpyruvate (PEP)-dependent systems (PTS): *levDEFG* encoding a complete mannose/fructose-specific enzyme IIABCD, *bglP* encoding a complete enzyme IIABC specific for beta-glucosides, two genes encoding the two subunits of BglH, a phospho-beta glucosidase, *mtlRA* coding for a PTS-dependent utilization of mannitol/glucitol, and *mdxK* encoding a maltose phosphorylase. *nagAB* encoding *N*-acetylglucosamine-6-phosphate deacetylase (EC 3.5.1.25) and *N*-acetylglucosamine-6-phosphate isomerase (EC 3.5.99.6) for *N*-acetylglucosamine degradation were both upregulated in CD337, while in TAP175, only *nagA* was upregulated. Two adjacent genes encoding a putative oligo-1,4-1,6-alpha-glucosidase (similar to the sucrase-maltase-isomaltase of *B. subtilis*) and MdxR, the transcriptional activator of the maltodextrin operon, and two others, located elsewhere in the chromosome and belonging to an *mdx* gene cluster, encoding a bifunctional beta-phosphoglucomutase/glucose-1-phosphate phosphodismutase (*mdxM*) and a putative IIBC component maltose PTS system, were specific of the TAP175 response. In addition, the gene encoding mannitol-1-phosphate dehydrogenase (*mtlD*) was upregulated in TAP175 only.

The regulatory pattern of the gene cluster encoding glycolytic enzymes (*cggR-gapA-pgk-tpiA-pgm-eno*) was strain dependent. Triose phosphate isomerase (TpiA, EC 5.3.1.1) and phosphoglycerate mutase (Pgm, EC 5.4.2.12) genes were upregulated in both strains whereas those encoding phosphoglycerate kinase (Pgk, EC 2.7.2.3) and enolase (Eno, EC 4.2.1.11) were upregulated in TAP175 only (LFC_2_ = 2.24 and 2.05 in TAP175 *vs.* 1.78 and 1.84 in CD337) and *cggR* only in CD337 (LFC_2_ = 2.11 in CD337 *vs.* 1.60 in TAP175). Moreover, a putative glycerate 2-kinase (GarK, EC 2.7.1.165) and a phosphoglycerate mutase were upregulated in both strains. Belonging to TAP175-specific response were a glucokinase encoding gene and *ppdK* encoding pyruvate phosphate dikinase. This enzyme catalyzes the reversible conversion of PEP + AMP + Pi into pyruvate + ATP (Lim et al., 2007). Lactate metabolism was upregulated in TAP175, with *lutC*, *lctE*, and *ldhL*. LutC is a protein involved in lactate utilization and the *B. subtilis lutABC* operon was reported as upregulated in stationary phase (İrigül-Sönmez et al., 2014). However, in *B. thermosphacta*, TAP175 *lutC* was the only gene of the operon to be upregulated. Two of the three paralogs encoding lactate dehydrogenase (EC 1.1.1.27), i.e., *ldhL* and *lctE*, were upregulated in TAP175. This enzyme catalyzes the reaction pyruvate <=> lactate and can lead, in association with *alsSD*, to the production of acetoin (Cruz Ramos et al., 2000). Interestingly, *alsSD* encoding acetolactate synthase (EC 2.2.1.6) and acetolactate decarboxylase (EC 4.1.1.5), respectively, which convert pyruvate to acetoin, were specifically upregulated in TAP175 with LFC_2_ values of 2.42 and 2.29 (whereas these values were only 1.24 and 1.38 in CD337). This correlates with previous results showing that TAP175 produced more acetoin than CD337 after growth in shrimp juice (Illikoud et al., 2019). However, in the present study, acetoin was not detected in the TAP175 VOC analysis from shrimp juice.

The upregulation of the abovementioned genes suggests an active carbon catabolism in shrimp juice and the utilization of a variety of sugars notably through the PTS system. However, carbon metabolism might be differentially efficient in the two strains. Indeed, as mentioned above, the central glycolytic gene repressor (*cggR*) was upregulated only in CD337. In *B. subtilis*, the regulatory function of CggR is dependent on fructose-1,6-bisphosphate (Zorrilla et al., 2007). Interestingly, *hprK* was also upregulated in CD337 (in TAP175 LFC_2_ was 1.88). It encodes the HPr kinase, which plays a central role in carbon catabolite repression. This enzyme possesses fructose-1,6-bisphosphate as an allosteric activator and is responsible for ATP-dependent phosphorylation of HPr, which itself regulates expression of catabolic genes and is required, when not phosphorylated by HprK, for the transport of PTS sugars (Jault et al., 2000). While *yvgN* encoding a methylglyoxal reductase was upregulated in both strains, *yhfP*, a gene coding for a putative NADP-dependent quinone oxidoreductase of unknown specificity, was upregulated in TAP175, and a putative hydroquinone-type dioxygenase/glyoxalase encoding gene (*mhqA*) was specifically upregulated in CD337. In *B. subtilis*, MhqA and YvgN have been proposed to be involved in the detoxification of methyl-glyoxal, a by-product of glycolysis (Töwe et al., 2007; Lei et al., 2009).

Amino acid metabolism was also modified depending on the growth substrate. The two genes *pdxT* and *pdxS*, encoding the two subunits of glutamine amidotransferase, were upregulated in shrimp juice. These are involved in pyridoxal 5′-phosphate biosynthesis and glutamine degradation. Eight genes of the gene cluster *hisIFAHBDGZ* involved in histidine biosynthesis were upregulated. However, *hisC* located at the 3′-end of the gene cluster and therefore putatively degraded was not upregulated. *gabD* encoding a putative succinate semialdehyde dehydrogenase that could be involved in amino acid or polyamine metabolism was upregulated in both strains. However, genes encoding a putative cystathionine beta-lyase/cysteine desulfhydrase and a D-alanine aminotransferase (*dat*) were specific of TAP175 response. The former enzyme (EC 4.4.1.8) produces L-homocysteine, an unstable compound that can lead to pyruvate production; the second (EC 2.6.1.21) catalyzes the reaction D-alanine + 2-oxoglutarate <=> pyruvate + D-glutamate. In addition, an aminopeptidase (PapA) and a tripeptidase (PepT) putatively capable of cleaving di- and tripeptides and the lysine transporter LysP were upregulated in TAP175.

Other genes encoding various functions were also upregulated by both strains but could not be associated to the functioning of whole pathways. Among those, *pyrKDFE* out of the *pyrRPBC(AA) (AB)KDFE* cluster were upregulated, meaning the synthesis of UMP from dihydro-orotate putatively induced in shrimp juice. Also, out of the *argCJBD* cluster involved in ornithine biosynthesis, only two genes (*argBD*) in CD337 and three (*argJBD*) in TAP175 were upregulated. *thiM* and *thiD* involved in thiamin metabolism were upregulated, but only in TAP175.

Concerning the strain-specific response to growth in shrimp juice, 19 genes encoding proteins of unknown function were upregulated in CD337 only, among which 6 were absent from the TAP175 genome sequence. Two miscellaneous RNAs were specifically upregulated in CD337. Three adjacent genes encoding a putative transposase, a putative recombinase resolvase, and one of the abovementioned protein of unknown function were specific of the CD337 response. Those belonged to a 24,132-bp genomic island specific of CD337 and putatively involved in cell surface or adhesion properties (Illikoud et al., 2018b).

A gene encoding a putative thioredoxin/glutaredoxin-like, which may act as an electron donor depending on the intracellular redox state (Petersohn et al., 1999), was upregulated in CD337. The genes encoding ribosome hibernation promoting factor hpf involved in the formation of inactive ribosomes during the stationary phase (Sato et al., 2009) and hbs, which may have an effect on gene expression through DNA binding (Klein and Marahiel, 2002), were upregulated in CD337 only. This may indicate a possible difference in the metabolic status of the two strains after 4 days of growth in shrimp juice. Finally, two genes encoding a putative immunity protein and a putative cation efflux protein were also specific of CD337 response but could not be linked to a specific metabolism or growth condition.

Conversely, except some genes discussed above, 45 were upregulated by TAP 175 only, among which 18 were of unknown function (with 8 absent or fragmented in the CD337 genome), 6 corresponded to RNAs (2 tRNAs, 4 misc RNAs), 1 corresponded to a putative prophage protein, and 5 corresponded to enzymes of unknown specificity.

Genes involved in general cellular machinery as translation, energy production, or DNA metabolism were upregulated specifically in TAP175. These encompassed genes encoding phenylalanyl-tRNA synthetase (*pheT*), D-Tyr-tRNATyr deacylase (*dtd*), translation initiation factor IF-2 (*infB*) and *atpF*, *atpA*, *atpG*, and *atpD* belonging to the *atpBEFHAGDC* operon encoding the F0F1-type ATP synthase. Five adjacent genes, i.e., *polA* encoding DNA polymerase I; *mutM* encoding formamidopyrimidine-DNA glycosylase, which prevents DNA damage by reactive oxygen species (Gómez-Marroquín et al., 2015); *coaE* encoding dephosphocoenzyme A kinase (E.C. 2.7.1.24), which catalyzes the final step of coenzyme A (CoA) synthesis; and two putative helicases similar to DnaB and DnaI involved in chromosome replication initiation, were upregulated only in TAP175. In addition, upregulation of *uvrA*, *mutL*, and *addA* involved in DNA damage recognition and DNA mismatch repair during chromosome replication (Cromie, 2009; Guarné and Charbonnier, 2015; Gómez-Marroquín et al., 2016) was observed only in TAP175. *gpsA* involved in phospholipid synthesis as responsible for sn-glycerol 3-phosphate synthesis (Morbidoni et al., 1995) was upregulated only in TAP175.

The catalase encoding gene *katA* was also upregulated only in TAP175. This enzyme has an antioxidant function by degrading hydrogen peroxide. Three CDS corresponding to a fragmented *trxB* gene were upregulated in TAP175. The fact that the three gene fragments were upregulated suggests sequencing errors (TAP175 genome sequence is a draft). *trxB* encodes thioredoxin reductase, which acts with thioredoxin (encoded by *trxA*) as an oxidoreductase system with antioxidant and redox regulatory functions. However, *trxA* was not expressed. As this gene is very short (318 nt), we cannot exclude that its expression could not be detected.

Finally, three putative regulators were specifically upregulated in TAP175: a putative stress-mediated DNA-binding transcriptional regulator whose gene is located immediately upstream from *hprK* in TAP175, but separated from it by a gene encoding a membrane protein of unknown function in CD337; yclK, a two-component sensor histidine kinase, whose gene is regulated by oxygen limitation in *B. subtilis* (Härtig et al., 2004); and a putative anti-sigma factor located downstream from a gene similar to *B. subtilis* genes encoding extracytoplasmic function (ECF) RNA polymerase sigma factors involved in the response to various stress or environmental conditions.

## Conclusion

This study provided insights into the relative difference in gene expression and VOC production by *B. thermosphacta*, during growth in meat or shrimp juice. We observed that *B. thermosphacta* metabolism was different on the two juices tested. The catabolism of meat or shrimp specific substrates may lead to different metabolisms depending on the food matrix. This may suggest that in the *B. thermosphacta* spoilage process, the biochemical composition of the food matrix drives the VOC production. The type of carbon source used by *B. thermosphacta* in laboratory medium has been reported to influence the production of spoilage molecules (Dainty and Hibbard, 1980). As an example, the production of acetoin was increased in the presence of glycerol, compared to ribose. Similarly, in our study, the utilization of different carbon sources, suggested by the expression of different sugar transporters in meat or shrimp juice, may affect the production of carbon-derived VOCs. Moreover, *B. thermosphacta* strains produced six detectable VOCs in shrimp juice, while only two were detected in meat juice (ethanol and acetoin). This suggests that *B*. *thermosphacta* alone most probably exerts a moderate effect on meat spoilage, since the human odor threshold is much lower for diacetyl than for acetoin (Casaburi et al., 2015). Nevertheless, as *B. thermosphacta* has been associated with spoilage of natural meat products in several previous studies, one can hypothesize that it may need interactions with other bacterial species within the meat microbiota, to increase its involvement in the spoilage process of meat. Conversely, in shrimp juice, *B. thermosphacta* alone can produce various molecules associated with the spoilage. However, this does not exclude that interaction of this species with other microbiota members could also intensify its spoilage potential of seafood products, as it has been previously described on cooked peeled shrimps, where *B. thermosphacta* co-inoculated with *Carnobacterium maltaromaticum* produced a typical wet dog off-flavor, whereas neither species produce this off-flavor when inoculated separately (Laursen et al., 2006).

**FIGURE 6:**
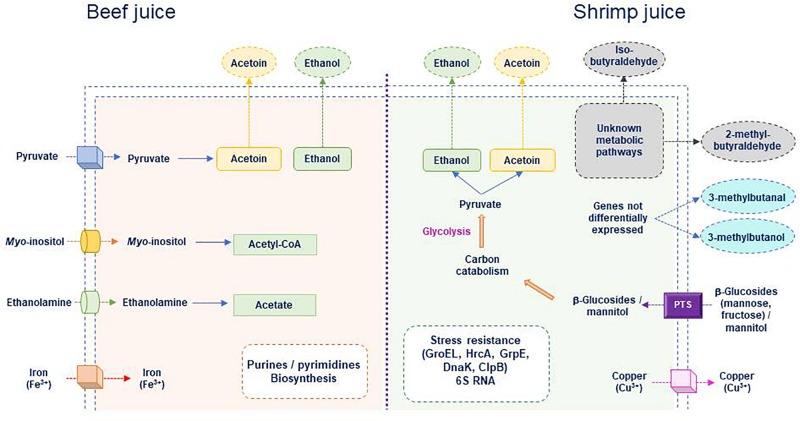
Main *B. thermosphacta* functions potentially induced during growth in meat or shrimp juice. Transport of molecules specifically present in meat or in shrimp for which transporter encoding genes are differentially expressed depending on the substrate is shown. Intracellular metabolic pathways specifically induced in shrimp or in beef juice are indicated as well as volatile organic compounds that were detected.

To our knowledge, this study is the first transcriptomic analysis performed to elucidate the genetic functions involved in the food spoilage process by *B. thermosphacta.* Figure 6 summarizes the main functions that could be specifically induced during growth in meat or shrimp. Two carbon sources (ethanolamine and *myo*-inositol) could be preferentially used in beef, whereas a larger panel of sugars could be transported, mainly through PTS systems, from shrimp juice. Iron uptake in meat and copper excretion in shrimp seemed to be induced. Amino acids, purine, and pyrimidine metabolisms appeared to vary depending on the food matrix but also on the strains. Physiological status also seemed different in meat and in shrimp (where stress response was upregulated). However, correlating the upregulated metabolic pathways to the production of molecules reported as associated to meat or shrimp spoilage was uneasy. This may result from the fact that transcriptome was analyzed at day 4 only. Further studies monitoring the dynamics of the transcriptomes during and after growth may provide more information.

## Data Availability Statement

The datasets generated for this study can be found in the Sequence Read Archive database, BioProject accession number PRJNA546512.

## Author Contributions

NI, DW, EJ, and MZ contributed to conception and design. NI, RG, CB, and DR acquired the data. NI, DW, DR, and MZ analyzed the data. NI and MZ drafted the manuscript for important intellectual content. All authors reviewed and revised the manuscript.

## Conflict of Interest

RG and DW were employed by the company Aérial. The remaining authors declare that the research was conducted in the absence of any commercial or financial relationships that could be construed as a potential conflict of interest.

## Supplementary Material

TABLE S1 Summary of RNA-seq data.

TABLE S2 Genes differentially expressed by *B. thermosphacta* CD337 and TAP175 when grown in beef or shrimp juice. Genes upregulated in beef compared to shrimp are detailed in the 1st sheet, those upregulated in shrimp juice compared to beef juice are detailed in the 2nd sheet. Genes upregulated by both strains are shown as well as genes specifically regulated in CD 337 or in TAP 175. Gene label, gene name and EC number of the encoded protein when available, are issued from genome annotation. The CD 337 complete genome was automatically annotated then manually cured (Illikoud et al., 2018b). For TAP 175 draft genome, cured annotations of CD 337 genome were transferred on the orthologous genes. For TAP 175 specific genes, initial automatic annotations were kept. Log Fold Change >2 are indicated. A color code indicates the level of upregulation.
