# Supplementary material for: Transcriptome and Volatilome Analysis During Growth of Brochothrix thermosphacta in Food: Role of Food Substrate and Strain Specificity for the Expression of Spoilage Functions
Source: Front Microbiol. 2019 Nov 8;10:2527. doi: 10.3389/fmicb.2019.02527 (PMC6856214; doi:10.3389/fmicb.2019.02527)
Supplement: TABLE S1 — Summary of RNA-seq data. [file Table_1.DOCX]

Supplementary Table 1 Summary of RNA-seq data.

|  | **Samples** | **Total number**  **of reads** | **Reads mapped on rRNA** | **Analyzed**  **reads** | **% of analyzed**  **reads** |
| --- | --- | --- | --- | --- | --- |
| **Shrimp juice** | CD 337-Replicate-1 | 17,994,011 | 656,829 | 16,104,771 | 89,5 |
|  | CD 337-Replicate-2 | 27,868,431 | 43,583 | 26,341,984 | 94,52 |
|  | CD 337-Replicate-3 | 16,591,350 | 27,217 | 15,977,713 | 96,3 |
|  | Average CD 337 | 20,817,931 | 242,543 | 19,474,823 | 93,55 |
|  | TAP 175-Replicate-1 | 22,909,377 | 96,881 | 21,736,762 | 94,88 |
|  | TAP 175-Replicate-2 | 21,142,474 | 95,607 | 19,315,917 | 91,36 |
|  | TAP 175-Replicate-3 | 20,486,747 | 122,987 | 18,834,959 | 91,94 |
|  | Average TAP 175 | 21,512,866 | 105,158 | 19,962,546 | 92,79 |
| **Meat juice** | CD 337-Replicate-1 | 17,057,863 | 91,325 | 15,849,566 | 92,92 |
|  | CD 337-Replicate-2 | 22,063,054 | 20,768 | 20,643,613 | 93,57 |
|  | CD 337-Replicate-3 | 19,036,542 | 7,452 | 16,392,076 | 86,11 |
|  | Average CD 337 | 19,385,820 | 39,848 | 17,628,418 | 90,93 |
|  | TAP 175-Replicate-1 | 21,983,405 | 104,026 | 20,865,072 | 94,91 |
|  | TAP 175-Replicate-2 | 19,054,166 | 14,970 | 17,882,940 | 93,85 |
|  | TAP 175-Replicate-3 | 22,001,593 | 624,111 | 18,188,678 | 82,67 |
|  | Average TAP 175 | 21,013,055 | 247,702 | 18,978,897 | 90,32 |
|  | **Total** | 248,189,013 | 1,905,756 | 228,134,051 |  |
